# Direct Ras G12C inhibitors: crossing the rubicon

**DOI:** 10.1038/s41416-019-0499-1

**Published:** 2019-06-26

**Authors:** Colin R. Lindsay, Fiona H. Blackhall

**Affiliations:** 1 Division of Molecular and Clinical Cancer Sciences, University of Manchester, Manchester, UK; 2 Department of Medical Oncology, The Christie NHS Foundation Trust, Wilmslow Road, Manchester, M20 4BX UK; 3 Cancer Research UK Lung Cancer Centre of Excellence, London and Manchester, Manchester, UK

**Keywords:** Non-small-cell lung cancer, Targeted therapies, Cancer genetics, Tumour biomarkers

## Abstract

Despite its status as the most commonly mutated oncogene in cancer, Ras has long been considered ‘undruggable’. In 2019, we will see the first clinical trial results for direct mutant Ras inhibitors, a result of persistent cross-disciplinary research that has been informed by a number of previous clinical and biological failures.

## Main

For clinicians treating non-small-cell lung cancer (NSCLC), 2018 was peppered with successive high-impact publications that partnered presentations at AACR, ASCO, WCLC and ESMO; developments dominated by new treatment indications such as NTRK fusion, huge therapeutic progress in the application of immunotherapy and tyrosine kinase inhibitors, and a confirmation of clear benefits from low-dose CT screening programmes.^1^ It is difficult to imagine a lung cancer landscape more different to 5 years ago when the first phase 1 trial of pembrolizumab was being reported.

Amongst this myriad of practice-changing studies, the first direct inhibitors of mutant K-Ras ready for clinical use, AMG 510 and MRTX 849, were presented at the AACR-Ras and general AACR congress over the past 6 months, followed shortly afterwards by a first report of clinical efficacy at ASCO 2019.^2,3^ To put this development into context, *KRAS* was first described in 1983 and it has taken 35 years to reach this point, whereas identification of oncogenic *BRAF* mutations in 2002 was followed by an effective targeted drug in 2009. The main reasons for this delay are (i) Ras is a small GTPase, whose affinity for GTP exponentially exceeds that observed between kinases and ATP, and (ii) it is a small smooth protein, with no good ‘pockets’ for small molecules to hang on to.^4^

Direct Ras drugs have resulted in particular from a tireless academic pursuit using new insights on the structural biochemistry of mutant K-Ras to iteratively define lead compounds (ARS-853 and ARS-1620), their optimisation, and their in vivo activity.^5–7^ This success has guided Amgen and Mirati Therapeutics (whose K-Ras drugs are the first on the clinical scene) as well as many other pharma companies who are targeting the Ras pathway.

One crucial aspect of targeting mutant K-Ras is that the developed drugs are covalent inhibitors irreversibly targeted to the cysteine residue of mutant *KRAS G12C*. Preclinical work has already shown that, as expected, they will not work on other mutant Ras alleles such as *G12D* and *G12V*.^7^ The mutant *G12C* subtype is most common in NSCLC and thus typically associated with smoking-related C>A genetic transversions. In general, *KRAS* is the most frequently mutated oncogene in human cancer on account of its per-patient presence in common cancers, such as lung and colorectal adenocarcinoma. Its mutation is not ubiquitous across different cancer types, so it is difficult to imagine how these important drugs will prosper if they do not find success in the increasingly competitive landscape of stage IV lung cancer.

**Fig. 1:**
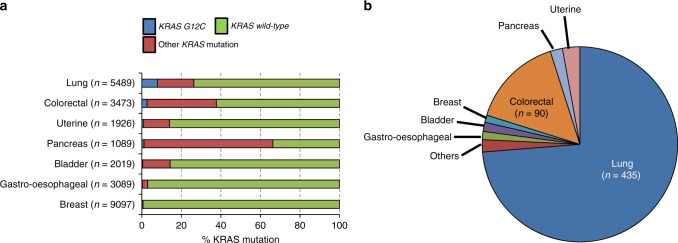
*KRAS G12C* mutation in cancer. **a** Proportions of *G12C* and non-*G12C KRAS* mutations in seven cancers with the most *KRAS G12C* cases. **b** Percentage contribution of each cancer type to *KRAS G12C* cancer

With all of the above in mind, we examined existing sequencing data on *KRAS*-mutant alleles, noting that we may finally be looking at an ‘actionable’ future for the large *KRAS* slice of the NSCLC molecular pie chart. A review of large-scale cancer sequencing programmes in cBioPortal confirmed that G12C was most prevalent in lung, colorectal and pancreas cancers, confirming KRAS mutation as a typical feature of recalcitrant epithelial tumours (Fig. 1a). Overall, NSCLC histology was most commonly associated with *KRAS G12C* mutant cases (70–75%), with colorectal cancer representing the main other significant proportion (Fig. 1b). For an expected raft of G12C drug trials in the coming years, we project that ~9,000–10,000 USA patients are diagnosed each year with stage IV *KRAS G12C* lung cancer.

How can success be optimised for G12C drugs in a molecular subset of cancer which is notorious for its unmet need? Beyond ensuring that drug pharmacokinetics and pharmacodynamics are appropriate, toxicity is anticipated to be a first small hurdle: *RAS* mutation should offer a therapeutic window, avoiding unwanted effects on healthy cells. Second, phase 3 trial design will be of crucial importance, a key lesson from the failed SELECT-1 phase 3 trial where the potential benefits of selumetinib in *KRAS*-mutant NSCLC were overestimated in an earlier phase 2 study.^8^ In the current NSCLC landscape, it is impossible to imagine a clinical line of sight that does not involve early assessment of combinations with checkpoint inhibitors. Toxicity in this setting may be of more concern, given the unpredictable side-effect profiles observed so far with dual immunotherapy/TKI treatments: while severe hepatic toxicity has been observed with nivolumab/crizotinib and pembrolizumab/gefitinib combinations, similar concerns have not been realised in early studies of alectinib/pembrolizumab and pembrolizumab/erlotinib.^9^ Third, if toxicity or efficacy does prove challenging in the first-line immunotherapy space, can RAS inhibitors establish their role as a monotherapy or combination optimised for a rapidly evolving second-line treatment landscape in NSCLC? Encouraging new phase I trial results from ASCO 2019 suggest that this should be the minimum target, with AMG 510 reporting a favourable toxicity profile and partial responses in 50% of NSCLC cases (5/10 patients, 1 PR unconfirmed).^10^

The application of these drugs to the minority of gastrointestinal cancers with G12C mutation could also still hold value. For example, colorectal cancer (CRC, ~4% with *G12C*) is the main cancer where upfront *KRAS* status is considered a prerequisite for treatment, with *RAS*/*RAF* mutation predicting lack of benefit from EGFR inhibitors such as cetuximab. The relative simplicity of identifying *KRAS G12C* in CRC is reflected by a phase I report showing that it was the main histological subtype recruited so far,^10^ although previous unexpected resistance to BRAF-mutant inhibition in CRC suggests reasons to be cautious. The dismal prognosis and paucity of treatment options for pancreas cancer (~2% with *G12C*) potentially confer a more amenable treatment landscape for breakthroughs to prosper, perhaps via recruitment with other *G12C* cancer types in pan-disease basket studies.

The Ras field has been here before. Other than selumetinib, failed studies of farnesyl transferase inhibitors offered a cautionary tale 10–15 years ago. Waterfall plots in early-phase trials should therefore be noted with a pause for further data, at least until survival advantages are confirmed by large-scale randomised trials. Most importantly, the iterative process of Ras research should not be deterred if these drugs do not succeed initially. On-treatment biopsies hold the key to understanding their mechanisms of resistance, which can inform subsequent drug development, clinical trials and research.

## Data Availability

The data appearing in Figure 1 were obtained from cBioPortal for Cancer Genomics (https://www.cbioportal.org/).^11^^,^^12^
